# Aortic pseudoaneurysm as a rare complication of purulent pericarditis: case report and literature review

**DOI:** 10.1186/s12872-025-05332-0

**Published:** 2025-11-24

**Authors:** Chun-Yu Yueh, Ming-Li Li, Ching-Feng Wu

**Affiliations:** 1 School of Medicine, China Medical University, Taichung, 404328 Taiwan; 2 Division of Cardiovascular Surgery, China Medical University Hospital, Taichung, 40447 Taiwan

**Keywords:** Purulent pericarditis, Aortic pseudoaneurysm, Methicillin-resistant *Staphylococcus aureus*, Delayed sternal closure, Thoracic endovascular aortic repair, Pericardiectomy

## Abstract

**Background:**

Purulent pericarditis has become rare in the antibiotic era, particularly when complicated by secondary infections such as an aortic pseudoaneurysm.

**Case presentation:**

We report a 46-year-old man who presented with persistent chest pain and cold sweats for three days. Imaging revealed a large pericardial effusion, and cultures grew methicillin-resistant *Staphylococcus aureus*(MRSA). The patient underwent partial pericardiectomy with delayed sternal closure and open irrigation. On day 23, he developed right shoulder pain, and imaging revealed a pseudoaneurysm of the ascending aorta. Thoracic endovascular aortic repair combined with bovine pericardial patch repair was performed. He survived and remained stable during a 13-month outpatient follow-up.

**Conclusion:**

Given the potential for fatal outcomes, clinicians should maintain a high index of suspicion and initiate prompt management, despite the rarity of this complication.

## Introduction

Purulent pericarditis has become uncommon in the antibiotic era, and its coexistence with an aortic pseudoaneurysm is even rarer. This combination can lead to a life-threatening clinical emergency with a high risk of fatal progression. However, the underlying pathophysiological connection remains unclear, as reported in several similar cases. Here, we describe a case of a 46-year-old man diagnosed with methicillin-resistant *Staphylococcus aureus* (MRSA) purulent pericarditis complicated by a mycotic pseudoaneurysm of the ascending aorta. To better understand this rare phenomenon, we reviewed 14 published cases [1–14], aiming to analyze their clinical features, explore plausible mechanisms, and provide a useful reference for future clinical practice.

## Case presentation

**Fig. 1:**
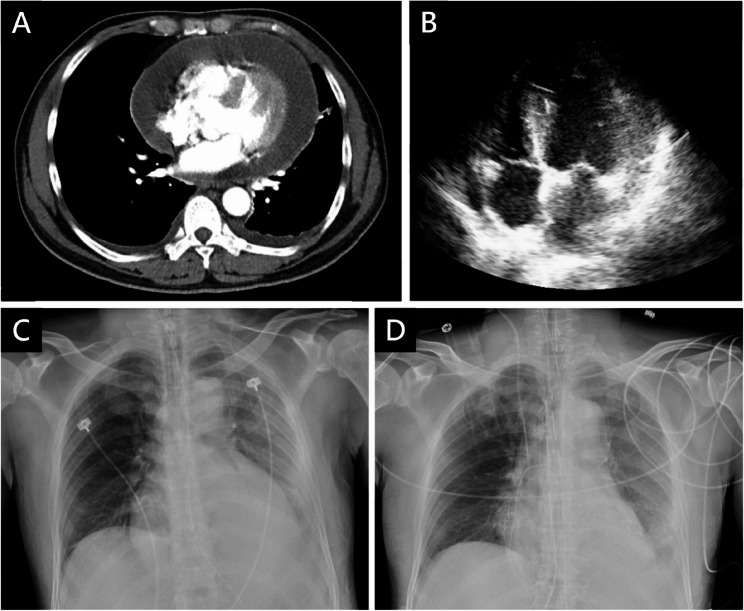
Preoperative Imaging of the Chest Upon Admission and Postoperative Radiograph. **(A)** Chest CT conducted at the former hospital revealed a 25.79 mm large pericardial effusion surrounding the entire heart. **(B)** TTE subcostal view showed a 25.48 mm pericardial effusion. **(C)** CXR (AP view) showed an enlarged heart configuration. **(D)** CXR (AP view) after partial pericardiectomy demonstrated a smaller heart size

**Fig. 2:**
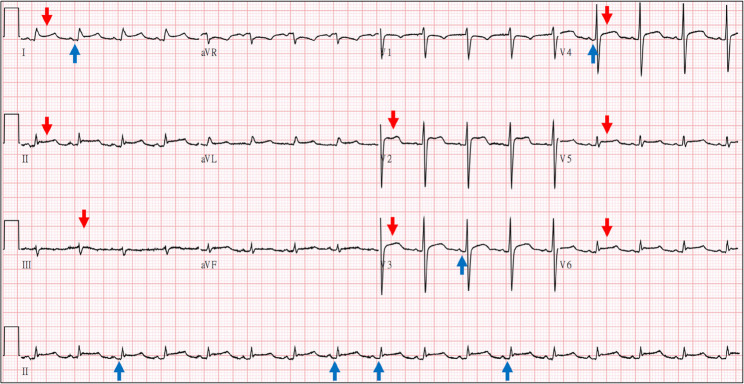
Electrocardiogram with Significant Pericarditis Patterns. The significant patterns included diffuse ST elevation *(red arrows)* and PR depression *(blue arrows)*

A 46-year-old man with a history of intravenous heroin use and hepatitis C was transferred to our medical center due to persistent chest pain and cold sweats for three days. At the referring local hospital, chest computed tomography (CT) and transthoracic echocardiogram (TTE) revealed a large pericardial effusion encasing the heart (25 mm; Fig. 1A, B). Pericardiocentesis (PCC) yielded 300 mL of yellowish, turbid fluid. Upon arrival at our emergency department, he exhibited tachycardia and tachypnea (temperature 36.0 °C, heart rate 102 bpm, blood pressure 141/104 mmHg, and respiratory rate 30/min). The electrocardiogram (ECG) demonstrated diffuse ST-segment elevations with PR segment depressions (Fig. 2). Chest X-ray (CXR) revealed an enlarged cardiac silhouette (Fig. 1C), and laboratory investigations showed leukocytosis (23,900/µL) and an elevated C-reactive protein (CRP 15.86 mg/dL). Empirical vancomycin plus ceftriaxone therapy was initiated based on the working diagnosis of purulent pericarditis.

**Fig. 3:**
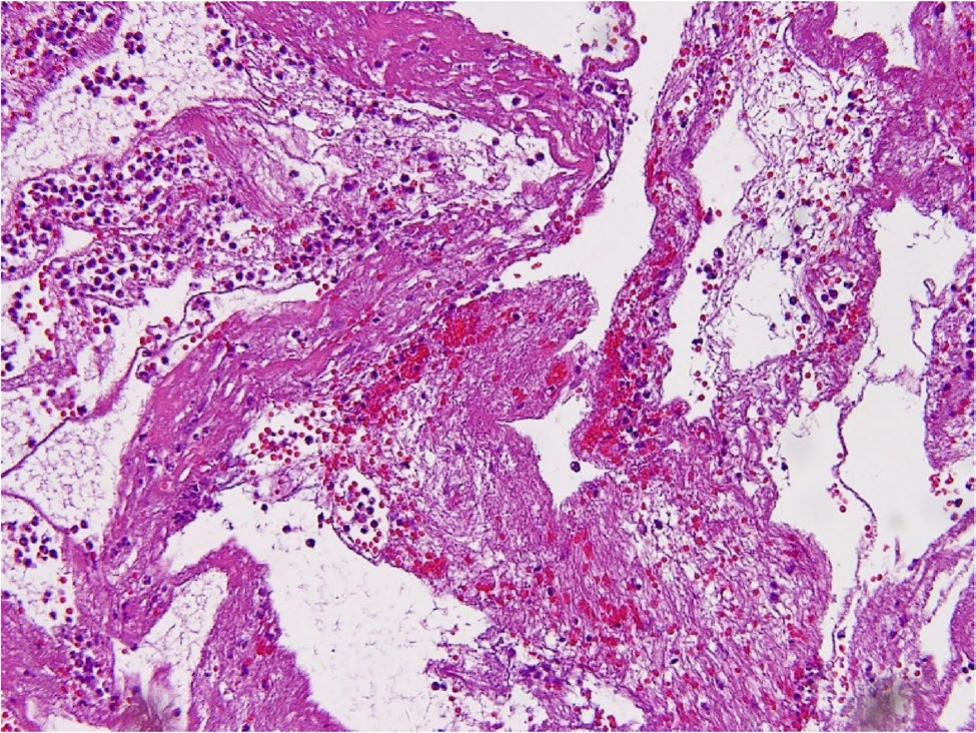
Microscopic Examination of Pericardium Specimen. Hematoxylin and eosin staining of the pericardium demonstrated suppurative inflammation with dense neutrophilic infiltration and fibrinous tissue

On day 2, partial pericardiectomy was performed to control the infection and prevent progression to constrictive pericarditis. Intraoperatively, 300 mL of purulent fluid was drained, and the pericardial cavity was irrigated with warm saline irrigation and packed with gauze. The surgery was uneventful, and the mediastinal wound was left open for ongoing observation and serial irrigation. The postoperative CXR demonstrated a reduction in cardiac size (Fig. 1D). Deep pus collected intraoperatively grew methicillin-resistant *Staphylococcus aureus* (MRSA), prompting escalation of antibiotics to daptomycin. Histopathological examination demonstrated suppurative inflammation with dense neutrophilic infiltration and fibrinous tissue (Fig. 3). Serial wound irrigations were performed every other day, and the sternal wound was closed on postoperative day 6.

**Fig. 4:**
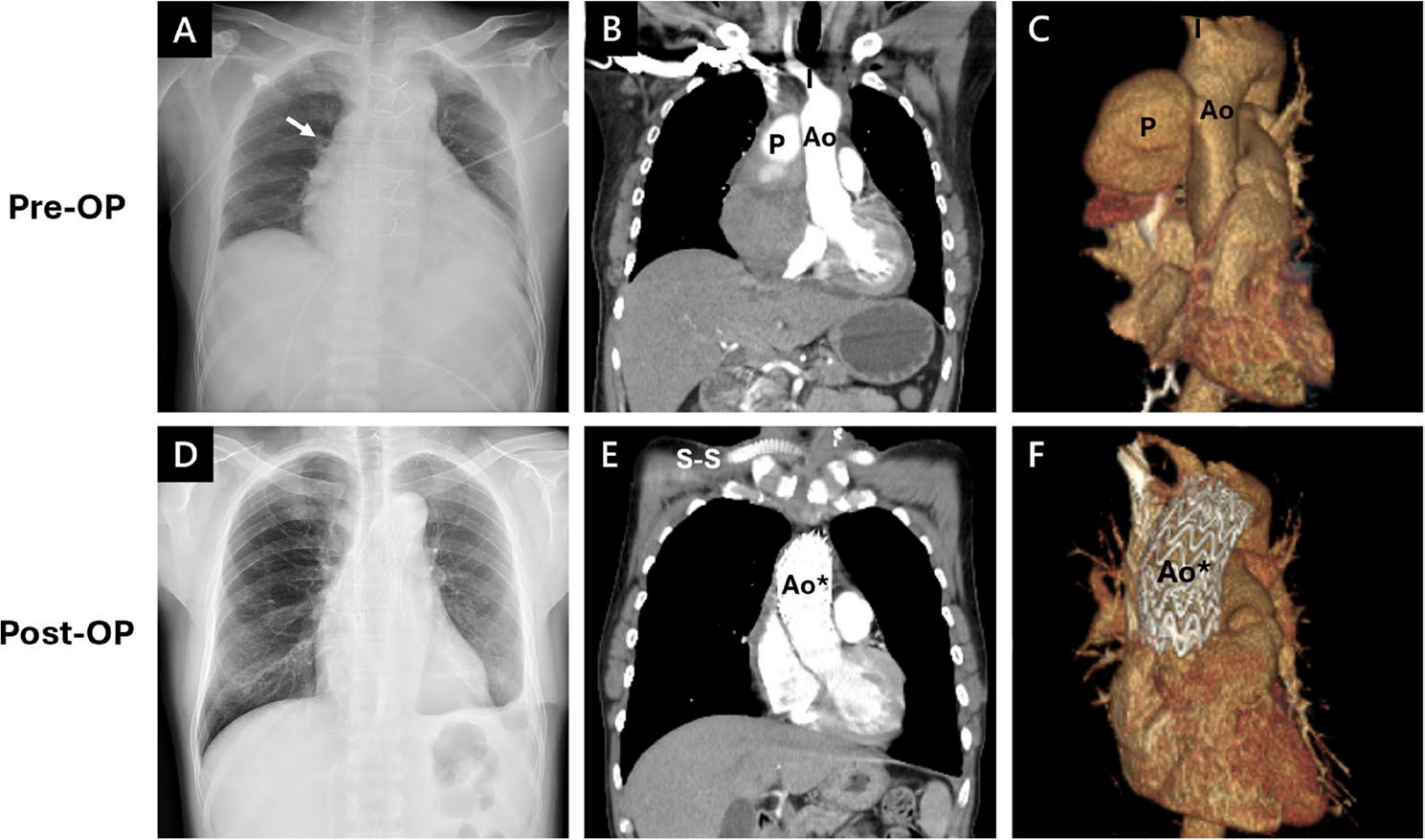
Imaging Studies Before (A-C) and After (D-F) Thoracic Endovascular Aortic Repair. (Ao, Aorta; I, Innominate artery; P, Pseudoaneurysm; Ao*, Aorta with TEVAR)

However, on day 23, the patient developed chest pain in the morning. His vital signs were within normal limits except for elevated blood pressure (145/113 mmHg). Five hours later, the pain radiated to his right shoulder and arm, for which diclofenac gel was prescribed for pain relief. Two hours afterward, the symptoms recurred, prompting further evaluation. Repeat vital signs revealed hypotension (88/53 mmHg) and bradycardia (58 bpm). The ECG showed normal sinus rhythm without abnormal patterns. Portable CXR demonstrated an enlarged cardiac configuration (Fig. 4A). Urgent CT scan revealed a contained rupture on the right side of the ascending aorta, forming a hematoma measuring 12.4 cm × 9.2 cm and compressing the superior vena cava (Fig. 4B and C). Upon recognition of this unexpected finding, the patient was immediately transferred to the operating room for emergency intervention.

**Fig. 5:**
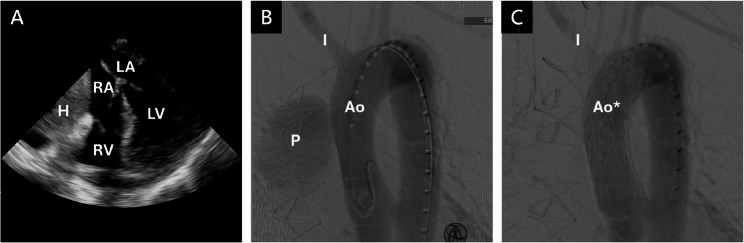
Transesophageal Echocardiography and Angiography Images of the Aortic Pseudoaneurysm. **(A)** TEE showed that the right atrium and right ventricles were compressed by the huge hematoma. **(B**,** C)** Angiography before and after TEVAR. *(H*,* Hematoma; RA*,* Right atrium; RV*,* Right ventricle; LA*,* Left atrium; LV*,* Left ventricle; P*,* Pseudoaneurysm; Ao*, Aorta; Ao*,* Aorta with TEVAR; I*,* Innominate artery)*

In the operating room, transesophageal echocardiography (TEE) confirmed a large hematoma compressing the right heart (8.03 × 6.34 cm; Fig. 5A). Thoracic endovascular aortic repair (TEVAR) was performed; partial occlusion of the innominate artery ostium necessitated a left-to-right subclavian artery bypass (S-S bypass) using an 8 mm × 40 cm vascular graft (Fig. 5B, C). Median sternotomy was then performed for debridement, and a 3 × 2 cm defect in the ascending aorta was repaired with bovine pericardium. Subsequently, a left anterolateral thigh (ALT) flap was utilized to reconstruct the sternal wound defect. The procedures went uneventfully, and the patient was discharged on day 45 with oral trimethoprim-sulfamethoxazole and doxycycline.

Two months later, contrast-enhanced CT scan showed no evidence of pseudoaneurysm or aortic rupture. The most recent visit to our outpatient department was 13 months after discharge, showing no signs of recurrence or complications (Fig. 4D–F).

## Discussion

According to the 2025 European Society of Cardiology (ESC) guidelines [15], the primary management of purulent pericarditis requires aggressive intervention to reduce its high mortality risk. This includes pericardial drainage combined with intravenous antibiotics, as well as other strategies such as subxiphoid pericardiotomy with rinsing, intrapericardial thrombolysis using streptokinase, or pericardiectomy. In our case, after partial pericardiectomy, a delayed sternal closure (DSC) was performed to allow repeated irrigation and direct inspection. The open sternotomy site was temporarily covered with a sterile bag and a drain, with gauze changed every other day. Our literature research revealed that typical irrigation involves the use of indwelling drains inserted during pericardiotomy for normal saline flushing; however, DSC with repeated open-cavity wet dressing changes has not previously been reported. We hypothesize that this unconventional approach may have contributed to direct aortic injury, subsequently resulting in the ascending aortic pseudoaneurysm.

**Fig. 6:**
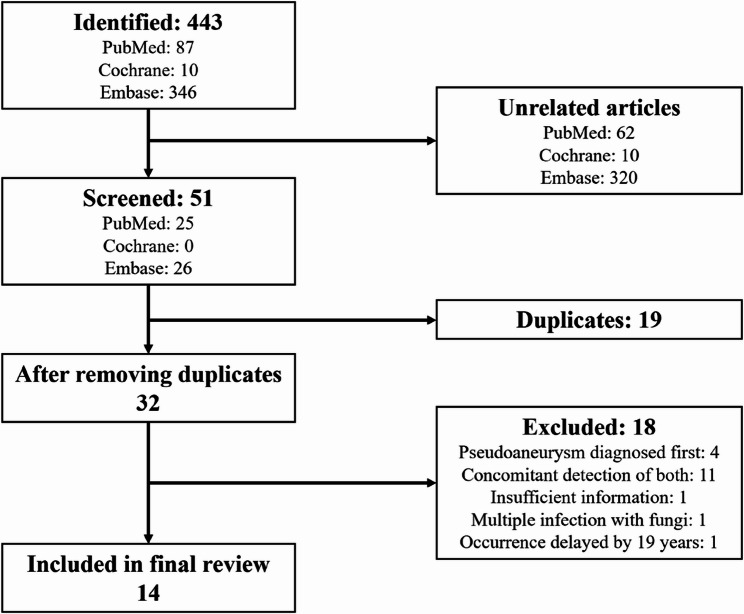
Literature Search Strategy and Screening Process

A systematic search was conducted using the keywords and the Boolean operators: (“Purulent pericarditis” OR “Pericardial effusion” OR “Pericarditis”) AND (“Pseudoaneurysm” OR “False aneurysm” OR “Mycotic aneurysm” OR “Infected aneurysm”) AND (“Aortic” OR “Aorta”). Because of the rarity of this complication, no time restrictions were applied, and only English-language publications were included. Three major medical databases – PubMed, Cochrane Library, and Embase – were searched and yielded 443 articles in total. After scanning abstracts, removing duplicates, and excluding irrelevant studies, 14 similar cases were included in the final review (Fig. 6).

Only 14 similar cases have been reported over the past 70 years. The underlying comorbidities included intravenous drug abuse, diabetes mellitus, and hypertension. *Staphylococcus aureus* (8 cases) and *Streptococcus pyogenes* (3 cases) were the predominant pathogens. Most patients underwent surgical interventions, including pericardial window, pericardiostomy, and pericardiectomy. Pseudoaneurysm occurred from immediately postoperatively up to day 91, and most commonly involving the ascending aorta, followed by the aortic arch and the sinus of Valsalva. Among these cases, 11 patients survived, whereas 3 expired before or after the diagnosis of pseudoaneurysm (Tables 1 and 2).

Pseudoaneurysm formation may result from one or more of the following mechanisms. The first is direct injury, which can occur secondary to previous invasive procedures; one article reported an infected aortic suture line after prior surgery as a causative factor [16], and other studies have shown that approximately 65% to 83% of patients with pseudoaneurysms had undergone a previous mediastinal operation [17–19]. All cases reviewed in our study involved one or more procedures before the development of the aortic pseudoaneurysm. The second factor is local extension; infection from adjacent sites – such as pneumonia or pericarditis – may extend to the aortic wall [16, 20–23]. All cases in this study involved pericarditis, suggesting that infection can possibly spread to the aorta in rare circumstances. The third factor is bacteremia in the presence of underlying aortic pathology, particularly atherosclerosis [11, 23], which is the most common condition in patients with pseudoaneurysm. Bacteremia may lead to microbial seeding of atherosclerotic plaques, causing progressive aortic wall erosion and eventual rupture. Other potential mechanisms, including lymphatic spread or septic embolization from infective endocarditis, have also been proposed to contribute to pseudoaneurysm formation.

**Table 1:** Summary of 14 reported cases of purulent pericarditis with pseudoaneurysm

	Author (Year)	Sex/Age	Chief Complaint	Medical History	Pathogen	Location of Pseudoaneurysm	Time
1	Foley MM (1956)	M/6	Febrile, malaise	-	*M. tuberculosis*	Sinus of Valsalva	91d
2	Fitzgerald JD (1964)	M/52	Lassitude, vague Ill	-	*S. aureus*	Ascending aorta	8d
3	Brahan RB (1990)	F/70	Substernal CP, fever	DM	*C. septicum*	Aortic arch	14d
4	Aranda J Jr (1998)	M/63	SOB, lightheadedness, fever, cough	PSVT	*S. aureus*	Ascending aorta	Post-OP
5	Barth H (2000)	F/16	SOB, fever, diarrhea	Varicella	*S. pyogenes*	Ascending aorta	2d
6	Ingoglia M (2011)	M/57	Substernal CP	HTN, HLD, CKD, COPD, smoking, CAD s/p PCI	*MSSA*	Ascending aorta	Post-OP
7	Erkut B (2011)	F/8	SOB, fever, sore throat	-	*Staphylococcus*	Ascending aorta	3d
8	Tan CO (2013)	M/38	RUQ pain	IVDA	*MRSA*	Sinus of Valsalva	Intra-OP
9	Han S (2016)	F/49	Severe SOB	Uncontrolled DM, HTN	*K. pneumoniae*	Ascending aorta	Post-OP
10	Meier D (2018)	M/42	Tiredness, diarrhea, jaundice, leg edema	IVDA, untreated HIV, HCV liver cirrhosis	*MSSA*	Ascending aorta	7d
11	Fry E (2018)	F/18	Pleuritic CP, SOB	Strep pharyngitis	*S. pyogenes*	Ascending aorta	A few days
12	Higuchi T (2022)	F/10	Neck and bilateral shoulder pain	-	*S. pyogenes*	Aortic arch	6d
13	Song JG (2023)	F/2	Persistent fever, AMS	-	*MRSA*	Ascending aorta	7d
14	Pandhair O (2023)	M/61	Positional CP, SOB	MRSA cellulitis	*MRSA*	Aortic arch	4d
*	This case (2025)	M/46	Persistent CP, cold sweat	IVDA, HCV	***MRSA***	Ascending aorta	21d

**Table 2:** Intervention of all reviewed cases of purulent pericarditis with subsequent pseudoaneurysm

	Pericardial Effusion/Volume (mL)	Intervention		Outcome
		Purulent Pericarditis	Mycotic Pseudoaneurysm	
1	Clear yellow/90	PCC, pericardiectomy	None (Autopsy)	Dead
2	Cloudy yellow/270; 3,000 in total (7 taps)	PCC, pericardial window	None (Ruptured)	Dead
3	Exudate/-	PCC	Aneurysmectomy + Dacron graft	Alive
4	Serosanguinous/750	PCC, pericardial window	None (Refused)	Alive
5	Honey-colored, viscous/150	PCC, pericardial window	Aneurysmectomy + homograft	Alive
6	Cloudy and bloody/-	Pericardial window (subxiphoid + left minithoracotomy)	Ascending aortic replacement (Hemashield graft) Closure of SVC fistula (Bovine pericardial patch)	Alive
7	Viscous/350	Subxiphoid pericardial drainage	Pericardial patch	Alive
8	Unmentioned	Pericardial window	Resection (anterior mitral valve leaflet homograft)	Alive
9	Yellowish, turbid/500	Subxiphoid pericardiostomy	Ascending aortic replacement (Hemashield graft)	Alive
10	Purulent/1250	Percutaneous drainage + subxiphoid surgical drainage	Ascending aorta + hemiarch replacement (Dacron graft)	Alive
11	Serous/300; Purulent/130	PCC + drainage	CorMatrix patch	Alive
12	Cloudy/200	Pericardial drainage	Aneurysmectomy + ascending aortic graft	Alive
13	Brownish-red with fibrin/330	PCC + catheter drainage + decortication	Aneurysmectomy + aortic repair	Alive
14	Hazy/600	PCC, pericardial window	None (Died before surgery)	Dead
*	Yellowish, turbid/300	PCC, partial pericardiectomy DSC, open drainage, wet dressing	Zone 0 TEVAR + Left-to-right S-S bypass Aortic repair (bovine pericardial patch)	Alive

**Fig. 7:**
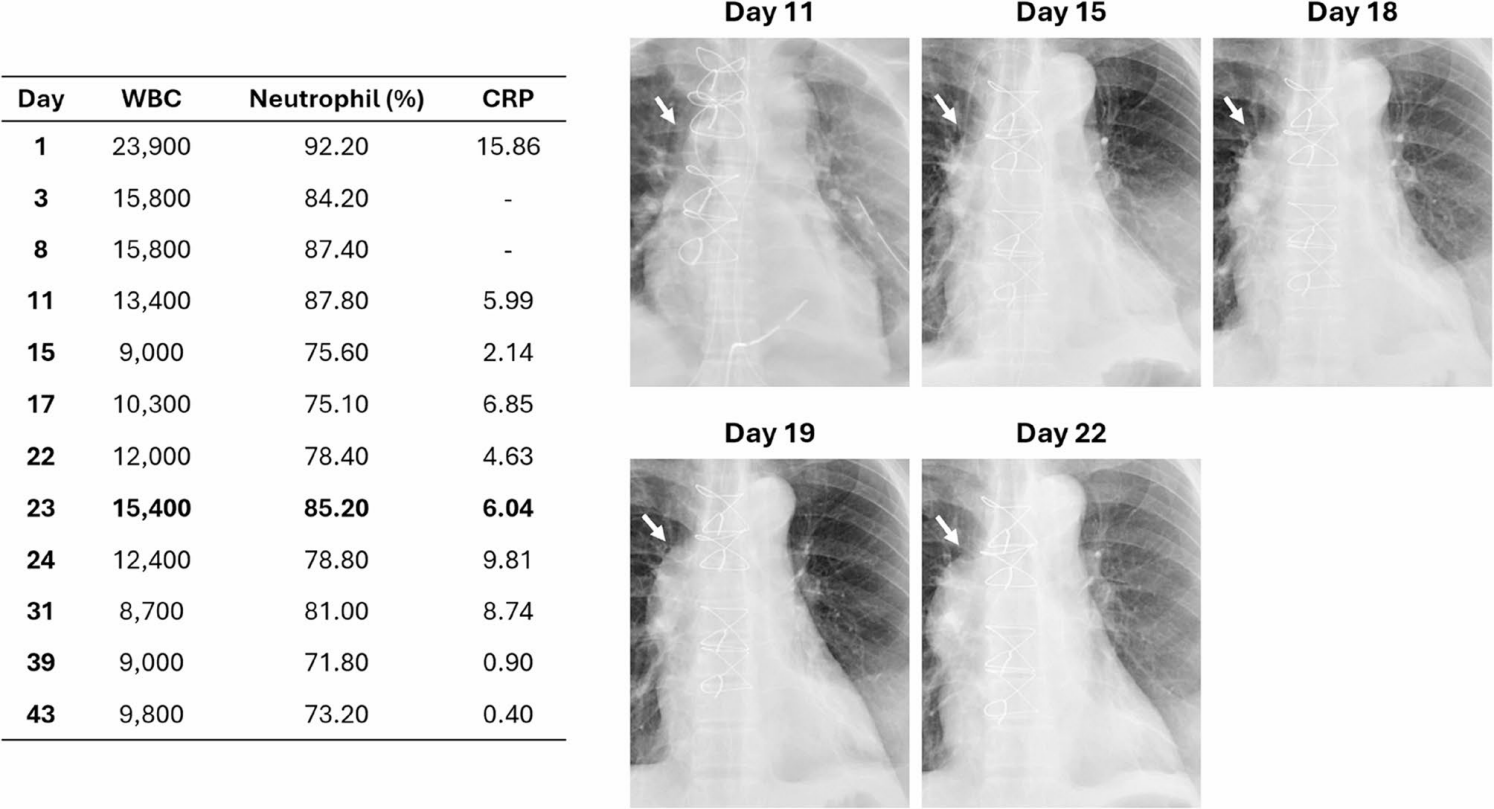
Serial Laboratory Data and Chest X ray Images Over the Hospital Course

In our patient, pseudoaneurysm formation was likely multifactorial. Although DSC with repeated wet dressings may have contributed to aortic wall injury, mechanical trauma alone seems insufficient to explain the delayed onset, as no pseudoaneurysm was identified during subsequent irrigations. The chest radiograph from day 11 to day 22 demonstrated a progressively enlarging right cardiac border, accompanied by elevated WBC and CRP levels, suggesting a potential infectious involvement of the ascending aorta (Fig. 7). Collectively, these findings indicate a probable association among repeated invasive management, ongoing infection from pericarditis, and subsequent pseudoaneurysm formation.

This study contains several limitations. First, as a single-case report with atypical postoperative course, its generalizability is limited; therefore, our observations should be interpreted as an association rather than a causal relationship. Second, the number of comparable cases in the literature review is very small, and the initial management strategies were heterogeneous, making statistical analysis arduous. Third, our review was constrained by the rarity of the condition and by reliance on published case reports, which are susceptible to reporting bias. These limitations cannot be overcome at present, and larger case series or systematic reviews will be necessary to prove its causality in the future.

## Conclusion

Purulent pericarditis complicated by an ascending aortic pseudoaneurysm is an exceptionally rare but potentially fatal condition. In this case, the most plausible pathogenesis was multifactorial, involving direct aortic injury with contiguous infectious spread from methicillin-resistant Staphylococcus aureus (MRSA) pericarditis. This report also highlights that delayed sternal closure – previously unreported strategy in this setting – may be a risk factor and should therefore be applied with caution. We recommend avoiding unnecessary invasive procedures and ensuring close surveillance during the early postoperative period.

## Data Availability

All data and images obtained during this study are included in this article.

## Abbreviations

AF: Atrial fibrillation
AMS: Altered mental status
CAP: Community-acquired pneumonia
CKD: Chronic kidney disease
COPD: Chronic obstructive pulmonary disease
CP: Chest pain
CRC: Colorectal cancer
DM: Diabetes mellitus
DOE: Dyspnea on exertion
F: Female
GBS: Group B Streptococcus
GERD: Gastroesophageal reflux disease
HCV: Hepatitis C virus
HLD: Hyperlipidemia
HTN: Hypertension
IPF: Idiopathic pulmonary fibrosis
IVDA: Intravenous drug abuse
LAD: Left anterior descending artery
LLL: Left lower lobe
M: Male
MRSA: Methicillin-resistant Staphylococcus aureus
MSSA: Methicillin-sensitive Staphylococcus aureus
N/V: Nausea and vomiting
PE: Pulmonary embolism
PNA: Pneumonia
RUQ: Right upper quadrant
SCC: Squamous cell carcinoma
SOB: Shortness of breath
TB: Tuberculosis
UC: Ulcerative colitis
